# Consistent Inhibition of Cyclooxygenase Drives Macrophages towards the Inflammatory Phenotype

**DOI:** 10.1371/journal.pone.0118203

**Published:** 2015-02-13

**Authors:** Yi Rang Na, Yi Na Yoon, Dain Son, Daun Jung, Gyo Jeong Gu, Seung Hyeok Seok

**Affiliations:** Macrophage Lab, Department of Microbiology and Immunology, and Institute of Endemic Disease, Seoul National University College of Medicine, 103 Daehak-ro, Chongno-gu, Seoul 110-799, South Korea; Centre for Inflammation Research, UNITED KINGDOM

## Abstract

Macrophages play important roles in defense against infection, as well as in homeostasis maintenance. Thus alterations of macrophage function can have unexpected pathological results. Cyclooxygenase (COX) inhibitors are widely used to relieve pain, but the effects of long-term usage on macrophage function remain to be elucidated. Using bone marrow-derived macrophage culture and long-term COX inhibitor treatments in BALB/c mice and zebrafish, we showed that chronic COX inhibition drives macrophages into an inflammatory state. Macrophages differentiated in the presence of SC-560 (COX-1 inhibitor), NS-398 (COX-2 inhibitor) or indomethacin (COX-1/2 inhibitor) for 7 days produced more TNFα or IL-12p70 with enhanced p65/IκB phosphoylation. *YmI* and IRF4 expression was reduced significantly, indicative of a more inflammatory phenotype. We further observed that indomethacin or NS-398 delivery accelerated zebrafish death rates during LPS induced sepsis. When COX inhibitors were released over 30 days from an osmotic pump implant in mice, macrophages from peritoneal cavities and adipose tissue produced more TNFα in both the basal state and under LPS stimulation. Consequently, indomethacin-exposed mice showed accelerated systemic inflammation after LPS injection. Our findings suggest that macrophages exhibit a more inflammatory phenotype when COX activities are chronically inhibited.

## Introduction

Cyclooxygenase-1 and 2 (COX-1 and COX-2; also known as prostaglandin endoperoxide synthase-1 and 2, PTGS-1 and PTGS-2) play a central role in the inflammatory cascade by converting arachidonic acid (AA) to prostaglandin H_2_ (PGH_2_), which in turn is converted to bioactive prostanoids by specific terminal synthases [1]. COX-1 and COX-2 are considered constitutive and inducible COXs, respectively. COX-2 is highly inducible by inflammatory stimuli; thus, it has been traditionally considered the most appropriate target for anti-inflammatory drugs. Both COX isoforms have ∼60% sequence identity at the protein level and catalyze the conversion of arachidonic acid to PGH_2_. The kinetic constants of the two isoforms for this reaction are similar, and the PGH_2_ produced is metabolized further to PGE_2_, PGI_2_, PGF_2_, PGD_2_ and thromboxane A_2_ [2].

Non-steroidal anti-inflammatory drugs (NSAIDs) represent a group of compounds of various classes with two common features; the absence of a steroid-like structure, and properties such as analgesic, anti-pyretic, and anti-inflammatory activities [3]. These drugs primarily inhibit the activity of COX enzymes, and thereby affect the synthesis of prostaglandin signaling molecules, which are involved in a wide range of physiological processes other than inflammation [4]. Currently, NSAIDs are the most widely indicated drugs to relieve pain in various chronic inflammatory diseases, such as rheumatoid arthritis, osteoarthritis, cancer and tissue injury [3]. However, the exact role of COX in inflammation, especially in macrophages, remains controversal. Mizuno *et al*. reported that COX-2 expression was linked to wound healing and localized to monocytes and macrophages, which further indicated that NSAIDs might inhibit normal immune resolution/wound healing if used in chronic diseases [5]. In addition, COX-2–deficient mice are more susceptible to LPS-induced neuronal injury and exhibit increased microglia and astrocyte activation [6,7].

Macrophages play important roles in various inflammatory responses. Although these plastic cells are known to be involved in defense against pathogens, they also play a role in maintaining homeostasis by clearing cellular debris generated during tissue remodeling and rapidly and efficiently clearing cells that have undergone apoptosis [8]. These processes occur independently of immune-cell signaling, and the removal of ‘effete’ or apoptotic cells results in little or no production of immune mediators by unstimulated macrophages [9]. Contributing to sentinel trophic functions, they are present in virtually all tissues including bone (osteoclasts), alveoli (alveolar macrophage), central nervous system (microglial cells), connective tissue (histiocytes), gastrointestinal tract, liver (Kupffer cells), spleen and peritoneum [10]. Resident macrophages have unique characteristics such as wound healing with anti-inflammatory functions (compared with classically activated macrophages, which fight infection by means of their pro-inflammatory activities) [11]. In this regard, alterations of resident macrophage characteristics could affect tissue homeostasis or inflammatory diseases.

Although we previously examined etodolac-mediated macrophage activation [12], whether chronic COX inhibitor usage alters systemic macrophage phenotypes requires further investigation. In this study, we assessed the effects of long-term COX inhibitor usage on macrophages which are the best-characterized innate immune cells with a role in inflammation. We found that chronic COX inhibition accelerated macrophage immune responses. Thus, possible modifications of NSAID prescription practices should be considered; removing or reducing their use during periods of remission rather than their continuous application may improve their efficacy in controlling chronic inflammatory diseases.

## Materials and Methods

### Cell culture and reagents

Murine bone-marrow-derived macrophages (BMDM) were derived from 6–9-week-old female BALB/c mice, as described previously. Isolated bone marrow cells were cultured for 7 days at a density of 10^6^/ml in RPMI medium (Thermo, Waltham, Massachusetts, USA) supplemented with 10% FBS (Gibco, Carlsbad, California, USA), 10 units/ml penicillin, 10 μg/ml streptomycin and 2 mM L-glutamine (Gibco, Carlsbad, California, USA) (hereafter termed complete medium) at 37°C in a humidified atmosphere with 5% CO_2_. To enrich the macrophage population using M-CSF, we supplemented complete medium with 10% L929 murine fibrosarcoma cell line culture supernatants. NS-398 (COX-2–specific inhibitor) and SC-560 (COX-1–specific inhibitor) were obtained from Cayman chemicals (Ann Arbor, Michigan, USA). Indomethacin was purchased from Sigma–Aldrich (St. Louis, Missouri, USA). All COX inhibitors used in this study were dissolved in DMSO. We treated COX inhibitors in two ways: Chronic exposure means that COX inhibitors were administered during the whole macrophage differentiation period with media changes at day 3, acute exposure means that COX inhibitors were treated just 30 minutes before LPS stimulation to already differentiated macrophages. For cytokine responses, macrophages were stimulated with LPS (100 ng/ml; *E.coli*, Sigma–Aldrich, St. Louis, Missouri, USA). NF-κB inhibitor was obtained from Santa Cruz Biotechnology (Dallas, Texas, USA).

### *In vivo* chronic COX inhibition and LPS stimulation

Female BALB/c mice were purchased from Orient Bio (Sungnam, Kyonggi, Korea). Mice were maintained under specific pathogen free conditions and cared for in the animal facility of Seoul National University Medical College. All animal procedures were performed according to the criteria outlined in the Guide for the Care and Use of Laboratory Animals prepared by the Institution of Animal Care and Use Committee of Seoul National University, Korea. The protocol was approved by the Seoul National University Institute Animal Care and Use Committee (Approval Number SNU-121005-3). Surgical procedures were performed under zoletil (Virbac, Carros, France) / xylazine (Bayer, Leverkusen, Germany) anesthesia, and all efforts were made to reduce unnecessary pain. For COX inhibitor release over 30 days, mice were implanted sub-cutaneously (s.c.) in the right scapular region using an osmotic pump (Alzet, Cupertino, CA, USA) carrying NS-398 (5 mg/kg/day), SC-560 (5 mg/kg/day) or indomethacin (1 mg/kg/day). To induce sepsis, mice were injected intraperitoneally (i.p) with 2.5 mg LPS per kg mice and sacrificed at the indicated time points. Blood was collected through a heart puncture under deep anesthesia and organs were sampled after cervical dislocation. For zebrafish, embryos 3 days after fertilization (dpf) were immersed with 1.25 μM NS-398 or 2.5 μM indomethacin for indicated time periods and stimulated with 50∼75 μg/ml LPS. Survival rates were evaluated at the indicated time points. Zebrafish larvae were maintained in sterilized Ringer’s solution at 28°C.

### *In vivo* macrophage preparation and cell culture conditions

Peritoneal cells were harvested from mice peritoneal cavities by washing with 10-ml RPMI. After centrifugation (5 min, 1300 rpm), cells were resuspended in RPMI complete medium. To obtain the macrophage population, we labeled cells with biotin conjugated F4/80 antibody (eBioscience, San Diego, CA, USA) and magnetic conjugated anti-biotin antibody (Miltenyi Biotec, Bergisch Gladbach, Germany). Positive selection was performed using an MS column (Miltenyi Biotec) according to the manufacturer’s instructions.

### Cytokine production

BMDMs were seeded in 96-well plates at 2×10^5^/200 μl density and cultured during 7 days. Culture supernatants were collected at 4 hours after LPS stimulation for TNFα or 24 hours for IL-12p70 and IL-10 detections. Isolated peritoneal macrophages were seeded in 24-well plates at 5×10^5^/ml and incubated for 2 h. Macrophages were stimulated with LPS (100 ng/ml) for 12 h, and supernatants were collected and stored at -80°C until analysis. TNFα, IL-12p70 and IL-10 concentrations were determined using the duoset ELISA kit (R&D systems, Minneapolis, MN, USA).

### Western blotting and antibodies

BMDMs were lysed with 1× sample buffer (Tris [pH 8], 2% SDS, 10 mM EDTA, 0.01% bromophenol blue, 25 mM 1,4-dithio-DL-threitol, and 250 mM 2-ME), heated to 95°C for 10 min and separated by 10% sodium dodecyl sulphate–PAGE. Proteins were then transferred to 0.45-μm PVDF membranes (Millipore, Billerica, Massachusetts, USA), and membranes were washed and blocked with 5% skim milk in PBS. Antibodies were incubated overnight at 4°C in 5% BSA 0.05% Tween-20, and 0.1% sodium azide PBS. Membranes were washed three times (10 min) before addition of secondary HRP-coupled antibody in 5% skim milk PBS for 1 h at room temperature. PICOlucent (Gbioscience, St Louis, MO, USA) was then added, and the film (Agfa, Septestraat, Mortsel, Belgium) was exposed and developed. Anti-phospho IκBα, anti-IκBα, anti-phospho p65, anti-p65, anti-IRF5 and anti-actin antibodies were purchased from Cell Signaling Technology (Beverly, MA, USA) and were used at 1/2000 dilutions. Anti-IRF4 antibody was obtained from Santa Cruz Biotechnology. The secondary polyclonal anti-mouse or -rabbit IgG HRP-coupled antibody was used at a 1/5000 dilution (Molecular probe, Eugene, Oregon, USA). Bands were quantitated and normalized with actin using ImageJ software.

### Quantitative real-time PCR

Total mRNA was extracted with the TRIzol reagent, according to the manufacturer’s instructions (Invitrogen, Carlsbad, CA, USA). cDNA was synthesized from 1-μg total RNA using M-MLT reverse transcriptase (Enzynomics, Daejon, Korea). Reverse-transcribed RNA (20 μg) was used as template for quantitative real-time PCR and set up in 96-well plates using Taqman Universal Mastermix II (Applied Biosystems, Foster City, California, United States). The primers and probe for *YmI* detection were: 5’-CATTGGAGGATGGAAGTTTGGA-3’ (Forward), 5’-GAATATCTGACGGTTCTGAGGAGTAGA-3’ (Reverse); 5’-CTGCCCCGTTCAGTGCCATGGT-3’ (probe). Gene expression levels were quantified using an ABI Prism 7900 sequence detection system (Applied Biosystems). The relative expression of *YmI* in each sample was normalized to GAPDH (Applied Biosystems) and compared with the controls according to the relative Ct method.

### Histology

Liver, lung and adipose tissues were resected, formalin-fixed, and paraffin-embedded using standard methods. Tissue sections were evaluated microscopically for inflammation indices by H&E staining.

### Statistical analyses

Student’s *t*-test, ANOVA and Log-rank Mantel-Cox test were performed to determine statistically significant differences between groups using GraphPad Prism (GraphPad Software, La Jolla, CA, USA). A *P*-value <0.05 was considered to indicate significance.

## Results

### Differentiated macrophages in the presence of COX inhibitors produce higher levels of inflammatory cytokines

**Fig 1:**
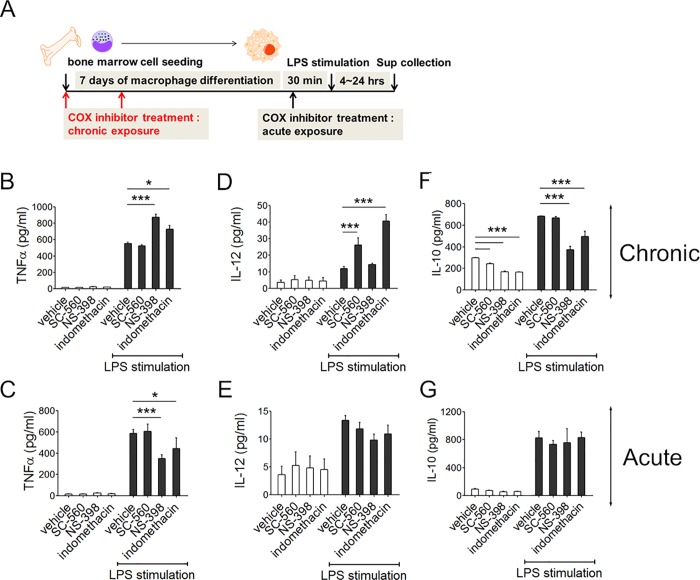
Cytokine responses in COX inhibited bone-marrow-derived macrophages. ***Panel A***: Time line of macrophage differentiation with COX inhibition and LPS stimulation. COX inhibitors were treated in two modes: SC-560 (COX-1 inhibitor), NS-398 (COX-2 inhibitor) or indomethacin (COX-1/2 inhibitor) were treated at 30 min before LPS stimulation (acute) or during whole differentiation period (chronic). ***Panels B-G*:** Macrophage TNFα (***Panels B, C***), IL-12p70 (***Panels D, E***) and IL-10 (***Panels F, G***) productions analyzed by ELISA. COX inhibitors were treated chronically (***Panels B, D, F***) or acutely (***Panels C, E, G***). LPS was treated at 100 ng/ml and culture medium was collected after 4 h (TNFα) and 24 h (IL-12p70 and IL-10) incubation for analysis. Results represent means ± SE of three independent experiments. Statistical analysis was performed by one-way ANOVA. *P<0.05, ***P<0.001.

**Fig 2:**
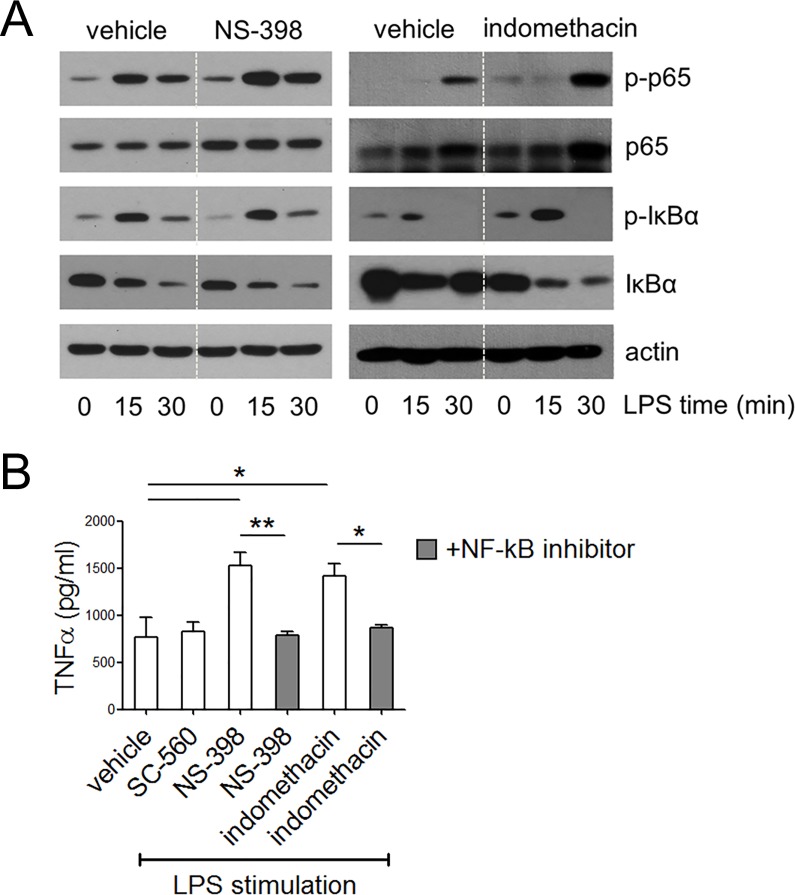
Analysis of TLR4 early signaling pathways in COX inhibited macrophages. ***Panel A*:** Phospho-IκBα, total IκBα, phospho-p65, total p65 and actin protein levels from whole cell lysates were detected by Western blotting. Macrophages were differentiated for 7 days in the presence of NS-398 or indomethacin and compared with the control at the indicated time points after LPS stimulation. Each compared lanes for one protein all come from same blots. ***Panel B*:** NF-κB inhibitor (20 ng/ml) was treated to chronically NS-398 or indomethacin treated macrophages 30 min before LPS stimulation. LPS was treated at 100 ng/ml and culture medium was collected after 4 h incubation for analysis. Results represent means ± SE of three experiments. Statistical analysis was performed by one-way ANOVA. *P<0.05, **P<0.005.

To investigate the effects of COX inhibition on macrophages, cultured bone marrow cells were exposed to various COX inhibitors, including the COX-1–specific inhibitor SC-560 (0.5 μM), COX-2–specific inhibitor NS-398 (50 μM) and COX-1/2 inhibitor indomethacin (100 μM). Timeline shows two modes of drug treatment (**Fig. 1A**); chronic exposure was started from bone marrow cell seeding and continued during 7 days of macrophage differentiation with fresh drug treatment at day 3. Acute exposure was started at 30 min before LPS treatment into the mature macrophages. When macrophages were stimulated with LPS, they produced significantly more TNFα in chronic NS-398 and indomethacin treated groups (**Fig. 1B**). Short-term 30 min pre-treatment of COX inhibitors before LPS stimulation reduced TNFα in both groups (**Fig. 1C**). In case of IL-12p70, SC-560 and indomethacin treated macrophages produced more amounts than control only when were exposed during whole 7 days of differentiation (**Fig. 1D, E**). Inversely, IL-10 secretion was lower in chronic NS-398 and indomethacin-treated macrophages (**Fig. 1F**). To enlarge basal state cytokine productions of macrophages, we investigated intracellular cytokine accumulation in F4/80^+^CD11b^+^ mature macrophages during 4 hours with Brefeldin A (**S1 Fig.**). FACS data clearly showed that chronic COX inhibitor treated macrophages synthesized more TNFα but less IL-10 compared with control in a basal state. Next, to confirm whether TLR early signaling pathway is relevant with TNFα production, we examined phospho-IκBα and phospho-p65 from whole cell lysates after LPS stimulation using Western blotting (**Fig. 2A**). Accordingly, both chronic COX-2 as well as COX-1/2 inhibited macrophages exhibited reduced total IκBα, as well as enhanced p65 phosphoylation upon LPS stimulation compared with the control. Enhanced NF-κB activity directly connected to TNFα production ability because enhanced TNFα productions were recovered when NF-kB inhibitor was treated before LPS stimulation in NS-398 and indomethacin groups (**Fig. 2B**). Collectively, these results indicated that macrophages lacking COXs activities during differentiation are more immune activated.

### Anti-inflammatory macrophage markers are reduced in COX inhibited macrophages

**Fig 3:**
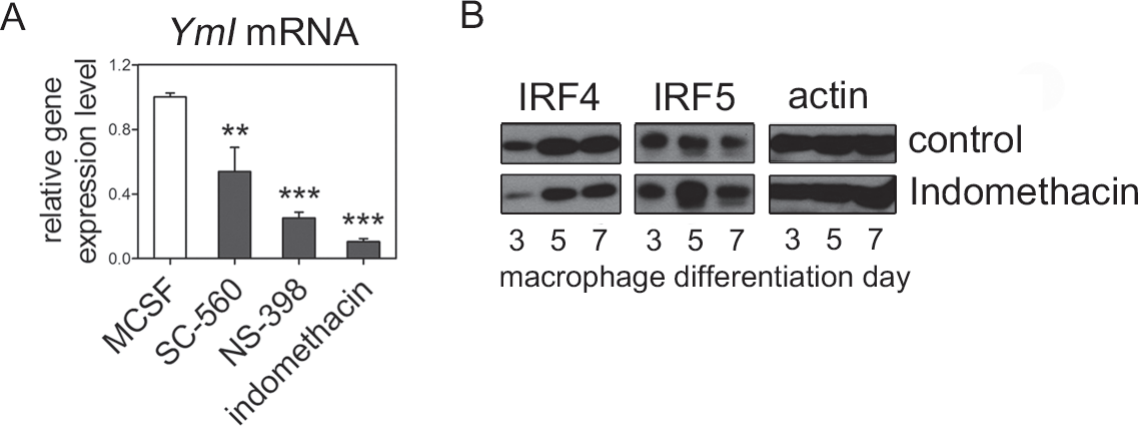
*YmI*, IRF4 and IRF5 expressions in COX-inhibited murine macrophages. Bone-marrow-derived macrophages were differentiated in the presence of indicative COX inhibitors for 7 days. ***Panel A*:** Real-time PCR was performed to examine *YmI* expression. Results represent relative fold changes compared with the control after normalization to endogenous *GAPDH* expression. Three independent experiments were performed. **P<0.01, ***P<0.001 ***Panel B*:** Western blotting representing IRF4, IRF5 and actin in macrophages at differentiation day 3, 5 and 7 with or without indomethacin. Result representative of three independent experiments.

We further examined whether intracellular functional macrophage markers were affected when COX activities were inhibited during macrophage differentiation. Three alternatively activated murine macrophage markers, *arginaseI, YmI* and *FizzI*, are often used to examine macrophage polarization states [13]. Thus, we analyzed the expression of these genes using real-time PCR. Among these, we found that *YmI* gene expression was significantly downregulated in COX inhibitor-treated macrophages (**Fig. 3A**). *Arginase I* and *FizzI* were not affected under our experimental conditions (data not shown). Other important macrophage markers are phenotype-determining transcription factors, including IRF4 and IRF5, which function during the early differentiation period and determine whether macrophages adopt the alternatively activated or classically activated phenotype, respectively [14]. Surprisingly, IRF4 was decreased but IRF5 was increased in indomethacin treated macrophages compared with control (**Fig. 3B**). Taken together, these results suggest that in the absence of COX activities, macrophages differentiate into more pro-inflammatory, classically activated phenotypes. Both COX-1 and COX-2 may be required for the anti-inflammatory properties of macrophages.

### COX inhibition accelerated LPS-induced zebrafish death rates

**Fig 4:**
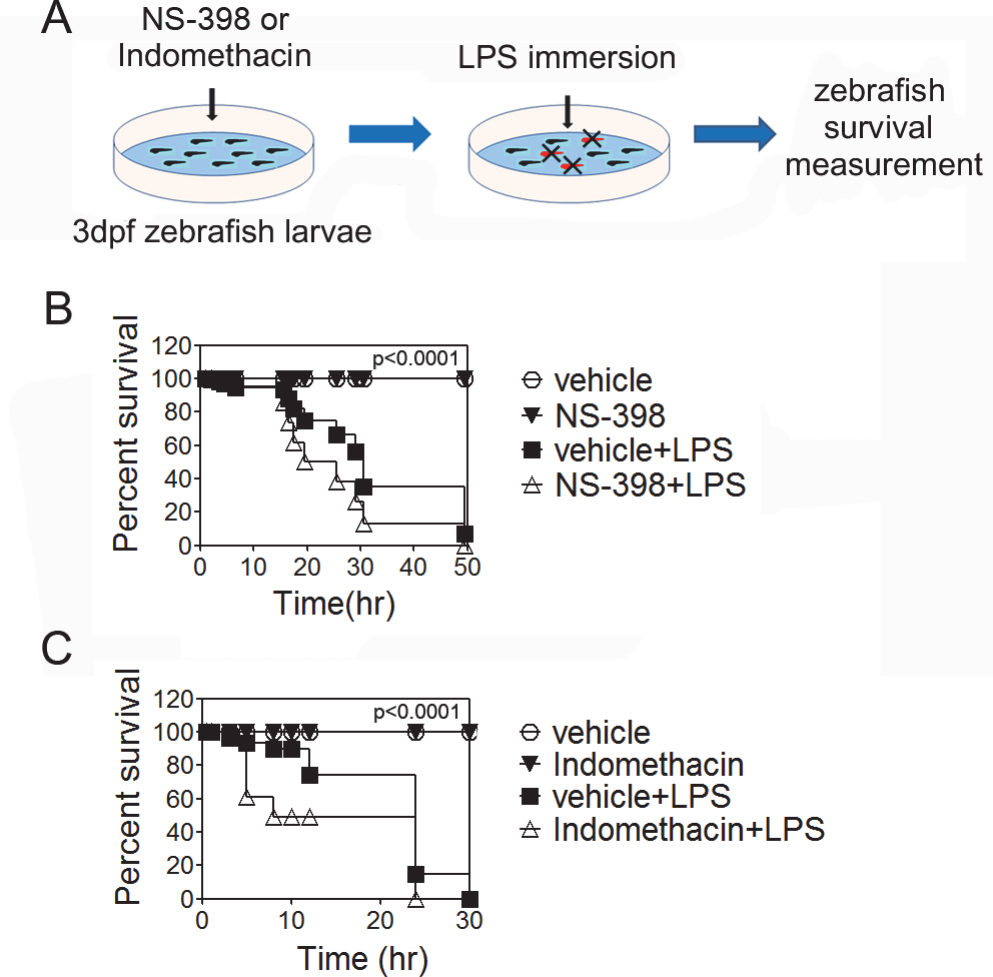
Survival curve of indomethacin-treated zebrafish larvae after LPS immersion. ***Panel A*:** A scheme of zebrafish sepsis model. Zebrafish larvae were maintained in Ringer’s solution containing 2.5 μM indomethacin or 0.01% DMSO from 3 to 8 days after fertilization (dpf). Solution and dead larvae were washed and fresh medium was added daily. After 5 days of indomethacin treatment, solution was changed and the larvae were immersed in 75 μg/ml LPS. ***Panel B*:** Survival curve of indomethacin treated zebrafish larvae in sepsis model. Data represents two independent experiments (n = 20). Statistical analysis was performed by Log-rank (Mantel-Cox) test between LPS Vs inhibitor+LPS groups. ***P<0.0001.

Macrophages are the leading cause of death associated with overproduction of pro-inflammatory cytokines in response to bacterial components. To explore the effects of COX inhibition on macrophage activity *in vivo*, we used zebrafish larvae, which have advantages as septic shock models in that they possess only innate immune systems until 7 days after fertilization, they are small and easily visible under a microscope, zebrafish larvae macrophages can produce TNFα and IL-1β similar to rodents [15], and they adhere to the animal welfare concept. Zebrafish larvae 3 days after fertilization were exposed to 1.25 μM NS-398 or 2.5 μM indomethacin and stimulated with a lethal dose (50∼75 μg/ml) of LPS (**Fig. 4A**). DMSO, NS-398 or indomethacin alone did not induce toxicity. During the observation period, NS-398 treated larvae showed rapid death rates by 20 h (75% versus 45%) compared to LPS alone group (**Fig. 4B**). Indomethacin-exposed larvae for five days showed more rapid death rates by 5 h (40% versus 5%) after LPS-induced sepsis, and all had died by 24 h (**Fig. 4C**). Without indomethacin, LPS induced a 25% death rate at 12 h and 100% at 30 h. These results suggest that macrophage activation might occur after COX inhibition *in vivo*.

### Long-term COX inhibition increases *ex vivo* macrophage production of TNFα upon LPS stimulation in mice

**Fig 5:**
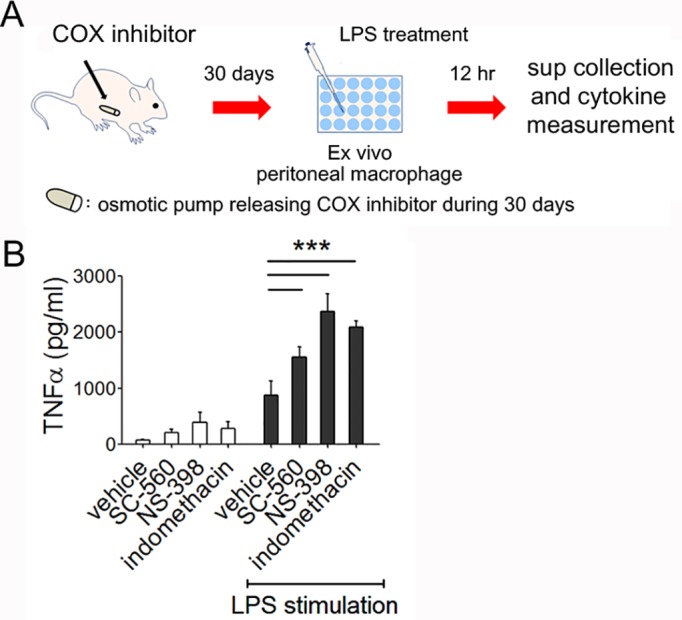
Ex vivo TNFα production by peritoneal macrophages of COX inhibitor-released mice. ***Panel A*:** BALB/c mice were anesthetized under zoletil/rompun and implanted with an osmotic pump (Alzet) releasing SC-560, NS-398, indomethacin or DMSO for 30 days. After 1 month, macrophages from peritoneal cavity were isolated using F4/80-positive cell magnetic sorting (Miltenyi Biotec) and cultured in 24-well plates with complete RPMI medium. Culture supernatant was collected after 12 h of incubation with LPS stimulation or in a basal state without LPS. TNFα levels were determined by ELISA. Results represent the means ± SE of five mice. Representative from two experiments. ***, *P*<0.001.

To explore long-term COX-inhibition-mediated macrophage activation, we administered drugs to BALB/c mice for 30 days using osmotic pump implantation (**Fig. 5A**). Optimal drug concentrations were selected based on animal weights, food consumption and the observation of bloody stool. The determined concentrations did not affect animal health during the 30 days of COX-1 or COX-2 inhibition (**S2 Fig.**). After 30 days of COX inhibition, we isolated F4/80-positive macrophages using a magnetic cell sorter from peritoneal flushing and cultured for 12 hours with LPS stimulation in 24-well plates. TNFα concentrations in culture supernatants were determined by ELISA. Macrophages apparently produced increased TNFα production in both COX-1 and COX-2 inhibited groups (**Fig. 5B**). These results collectively indicates that 30 days of COX inhibition *in vivo* could alter resident macrophage phenotypes into more inflammatory state.

### Long-term COX inhibition exacerbated LPS-induced systemic inflammation in mice

**Fig 6:**
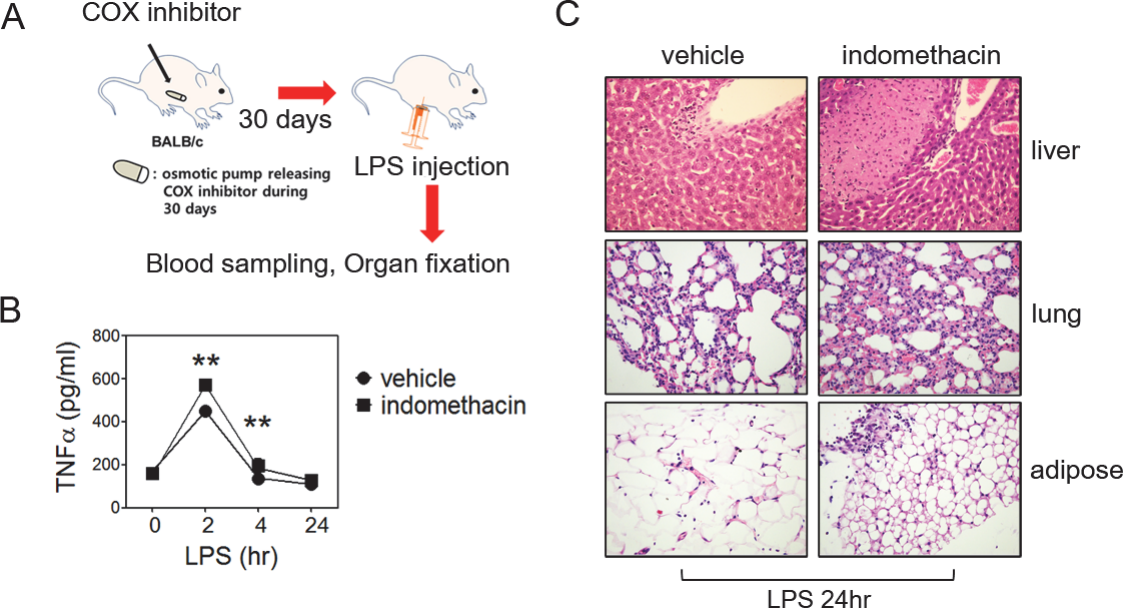
LPS-induced systemic inflammation of indomethacin-released mice. ***Panel A*:** A scheme of mouse sepsis model. ***Panel B*:** Time course serum TNFα concentrations of indomethacin-released mice. BALB/c mice after releasing DMSO or indomethacin for 30 days using osmotic pump were intraperitoneally injected with 2.5 mg of LPS per kg mouse. At indicated time points, blood was collected and serum TNFα levels were determined by ELISA. Results represent data from five mice. Statistical analysis was performed using Student’s *t*-test. **, *P*<0.01 ***Panel C*:** Histopathology of indomethacin released mice after LPS injection. LPS-injected mice were sacrificed after 24 h. Liver, lung and adipose tissue were fixed in 10% formalin. Formal alcohol–xylene series were processed and paraffin-embedded sections were stained with hematoxylin and eosin. Results are representative of five mice.

We next injected 2.5 mg of LPS per kg mouse into 30-days of indomethacin or DMSO released mice to induce systemic inflammation (**Fig. 6A**). At this LPS concentration, mice suffered from inflammation but were resolved within 72 hours. As expected, serum TNFα levels were continuously higher in indomethacin-treated mice (**Fig. 6B**) compared with control mice. Histopathology at LPS 24 hours showed more severe inflammation, including necrotic foci in the liver, interstitial pneumonia in the lung and leukocyte infiltration in adipose tissue in indomethacin-treated mice (**Fig. 6C**). These results demonstrate that long-term COX inhibitor treatment exacerbated systemic inflammatory response in BALB/c mice.

## Discussion

Our data suggest that chronic COX inhibition generates immune-enhanced macrophages. These macrophages produced more TNFα and less IL-10 in their naïve state. They were charged for activation upon LPS stimulation, and thus capable of synthesizing more TNFα or IL-12p70 after stimulation. Consequently, long-term COX inhibition *in vivo* resulted in systemic macrophage activation, which drives more severe inflammatory states after LPS challenge.

Previous studies have explored the effects of COX inhibition on macrophages [16–19]. However, the majority of these focused on the role of COX-2 in inflammation. Typically, COX inhibition was shown to mediate anti-inflammatory effects. Shackelford *et al*. reported aspirin-mediated suppression of TNFα gene transcription in primary murine macrophages [20] and concluded that aspirin exerts significant anti-inflammatory effects by suppressing the production of macrophage-derived inflammatory mediators. However, more recent studies indicated that COX inhibition mediates inflammatory responses. For example, macrophages without COX-2 showed enhanced bactericidal activity [19], increased phagocytic ability [21] or TNFα overproduction. These results are typically interpreted based on PGE_2_, because PGE_2_ has anti-inflammatory effects through the G-protein coupled EP receptor/cAMP/PKA signaling pathway [22]. The resultant NF-κB inhibition and CREB-mediated IL-10 production contributed to the overall macrophage anti-inflammatory function, which was blocked by COX inhibitors. In fact, one of our previous studies revealed that chronic COX inhibition in murine macrophages using indomethacin apparently reduced M2 marker gene expressions including *C/EBPβ, MrcI* and *IL-10* through autologous PGE_2_ inhibition (manuscript in preparing). It is also known to be associated with the immune resolution program by resolving E1 and protectin D1, which are blocked by COX-2 inhibition [23].

Our data revealed that chronic COX inhibition mediates macrophage differentiation into more inflammatory phenotypes, although short term inhibition did not. They produced more TNFα in both the naïve and LPS-stimulated states, with enhanced activation of NF-κB signaling pathways. IRF4 and *YmI* downregulation during macrophage differentiation under COX inhibition further indicate that COXs are required for adequate M2-type macrophage phenotype development. Consistent with our results, there are several reports about inflammatory cytokine production of immune cells after several days of NSAIDs treatment from healthy donors. After 3 days of oral aspirin treatment, whole blood from six healthy volunteers produced increased TNFα and IFNγ [24]. In another study, IL-1α induced IL-1β synthesis was elevated to a mean individual increase of 538% and synthesis of TNFα was elevated to 270% in peripheral blood mononuclear cells after daily ibuprofen treatment for 12 days and 2 weeks of discontinuation period in seven volunteers [25]. We still do not know whether monocytes/macrophages are involved in these reports as well as whether the serum cytokine profiles has been also changed because of long term NSAIDs treatment in healthy man, however these reports possibly support our findings about the roles of COX enzymes on innate immune cell polarization. In fact we observed that human monocyte derived macrophages produced increased TNFα, similar with the observations in our murine experiments, when COX-2 was inhibited during differentiation period [12]. Although exact mechanisms on human patients should be further examined, our data together with other reports collectively suggest possible innate immune enhancing effects of long term COX inhibition in human.

COX inhibition-mediated immune activation is better understood in cancer. Clinical observations suggest that indomethacin enhances macrophage-mediated host defense against tumors by inducing monocyte-derived secretion of interleukin (IL)-1 and tumor necrosis factor alpha (TNFα) in tumors [26]. Rofecoxib, a COX-2–specific inhibitor, is known to increase monocyte binding to intracellular adhesion molecule-1 (ICAM-1) with concomitant enhancement of tumor tissue infiltration and restoration of immune function in head and neck cancer patients [17]. These findings indicated that NSAIDs (particularly COX-2 inhibitors) enhance tumor-associated-macrophage (TAM)–mediated anti-tumor immune responses by increasing cytokine production from monocytes.

COX-1 is expressed constitutively in resident inflammatory cells, suggesting that COX-1 provides prostaglandins, which are required for homeostatic functions [27]. Interestingly, COX-1 contributes to the macrophage phenotype during bone-marrow-derived cell differentiation. The COX-1–specific inhibitor SC-560 functions in macrophages since chronic SC-560 treated macrophages produced more IL-12p70 (**Fig. 1D**). Because differentiating bone-marrow-derived macrophages express significant level of endogenous COX-1 (**S3 Fig.**), constantly produced prostaglandins might influence macrophage phenotype autologously. Meanwhile, there exists apparent effects of COX-2 on macrophage development in that NS-398 treated bone marrow derived macrophages increased their TNFα and reduced IL-10 secretion (**Fig. 1B, F**). We detected much lower COX-2 expressions compared to COX-1 in differentiating macrophages (**S3 Fig.**), thus the exact mechanism how chronic COX-2 inhibition in naïve macrophages could enhance LPS mediated NF-κB activation requires further investigation. Similar results were obtained using lung cancer model [28] in that chronic COX-2 inhibition reduced antigen presenting cell mediated IL-10 secretion but enhanced IL-12 secretion in the tumor microenvironment and consequently reduced tumor size. Although they showed that anti-PGE_2_ antibody could mimic COX-2 inhibitor effect in the tumor model, the specific prostaglandins in relation with the roles of COX-1/2 in differentiating resident macrophage phenotypes should be examined to understand endogenous macrophage physiology.

To evaluate the systemic consequences of COX inhibition in a pathophysiological state, we induced septic inflammation using LPS. Zebrafish larvae respond to LPS with systemic TNFα and IL-1β production [29]. In addition, COX-1/2 homologues in zebrafish macrophages were shown to be functional [30], and thus COX inhibitors could function in zebrafish embryos. Since it is simple to generate reproducible LPS survival curves, we treated zebrafish embryos with NS-398 or indomethacin, followed by lethal LPS stimulation. As expected, COX-2 as well as COX-1/2 inhibition increased zebrafish embryo death rates (**Fig. 4B, C**). Similar to zebrafish, mice subjected to long-term indomethacin treatment showed more severe inflammation in the liver, lung and adipose tissue after injection with sub-lethal doses of LPS (**Fig. 6C**). Serum TNFα was continuously higher in indomethacin treated mice after LPS challenge (**Fig. 6B**) and this overall exacerbated cytokine response might confer more severe inflammation in mice. Collectively, animal study results are in agreement with *in vitro* data, which showed enhanced TNFα secretion in differentiated bone marrow derived macrophages when COXs are inhibited (**Fig. 1**). Because macrophage phenotypes and activation potential in each organ affect disease severity and duration in acute inflammation such as sepsis [31], if long-term COX inhibitor intake could alter endogenous macrophage phenotypes, it would consequence accelerating inflammation in the presence of specific stimulants. Our data clearly support the concerns regarding the effects of COX inhibitors on macrophages.

In conclusion, we explored the role of COX in macrophages. Long-term COX inhibition drives macrophages into more inflammatory states, so it is important to determine the roles of COX in resident macrophage differentiation. Future human studies could increase our understanding of macrophage polarization and the effects of clinical NSAIDs on macrophage-mediated inflammatory diseases.

## Supporting Information

S1 Fig Basal cytokine production in COX inhibited bone-marrow-derived macrophages during differentiation. Bone marrow cells were obtained from the femurs of female BALB/c mice and differentiated during 7 days in RPMI complete medium with or without SC-560 (COX-1 inhibitor), NS-398 (COX-2 inhibitor) or indomethacin (COX-1/2 inhibitor). Macrophages were treated with 100 ng/ml Brefeldin A and intracellular cytokines were accumulated during 4 h starting from LPS 0 h (TNFα) or LPS 8 h (IL-12p40 and IL-10). Cells were scraped, fixed with 4% paraformaldehyde, and stained with appropriate antibodies. FACS analysis was performed in F4/80–CD11b double-positive populations. Results represent means ± SE of three independent experiments. Statistical analysis was performed by one-way ANOVA. *P<0.05, **P<0.01, ***P<0.001.
(TIF)

S2 Fig Mice weights after COX inhibitor release from osmotic pump implantation. Female BALB/c mice were anesthetized and implanted subcutaneously with an osmotic pump carrying DMSO, SC-560, NS-398 or indomethacin. Mice weights were recorded twice per week for 1 month. Means ± SE, n = 5.
(TIF)

S3 Fig COX-1 and COX-2 expressions in macrophages. Bone marrow cells (Day 0) and differentiating macrophages (Day 1, 3, 5, 7) were fixed with 4% PFA, permeabilized and stained with COX-1 and COX-2 antibodies. Mean fluorescence intensities were analyzed using FACS.
(TIF)
