# Supplementary figures and images for: Consistent Inhibition of Cyclooxygenase Drives Macrophages towards the Inflammatory Phenotype
Source: PLoS One. 2015 Feb 13;10(2):e0118203. doi: 10.1371/journal.pone.0118203 (PMC4334507; doi:10.1371/journal.pone.0118203)

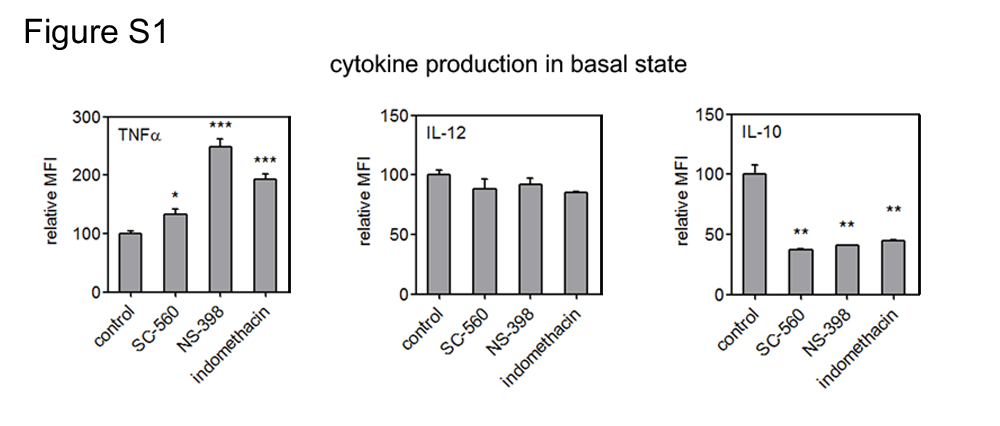

Supplement: S1 Fig — Bone marrow cells were obtained from the femurs of female BALB/c mice and differentiated during 7 days in RPMI complete medium with or without SC-560 (COX-1 inhibitor), NS-398 (COX-2 inhibitor) or indomethacin (COX-1/2 inhibitor). Macrophages were treated with 100 ng/ml Brefeldin A and intracellular cytokines were accumulated during 4 h starting from LPS 0 h (TNFα) or LPS 8 h (IL-12p40 and IL-10). Cells were scraped, fixed with 4% paraformaldehyde, and stained with appropriate antibodies. FACS analysis was performed in F4/80–CD11b double-positive populations. Results represent means ± SE of three independent experiments. Statistical analysis was performed by one-way ANOVA. *P<0.05, **P<0.01, ***P<0.001. (TIF) [file pone.0118203.s001.tif]

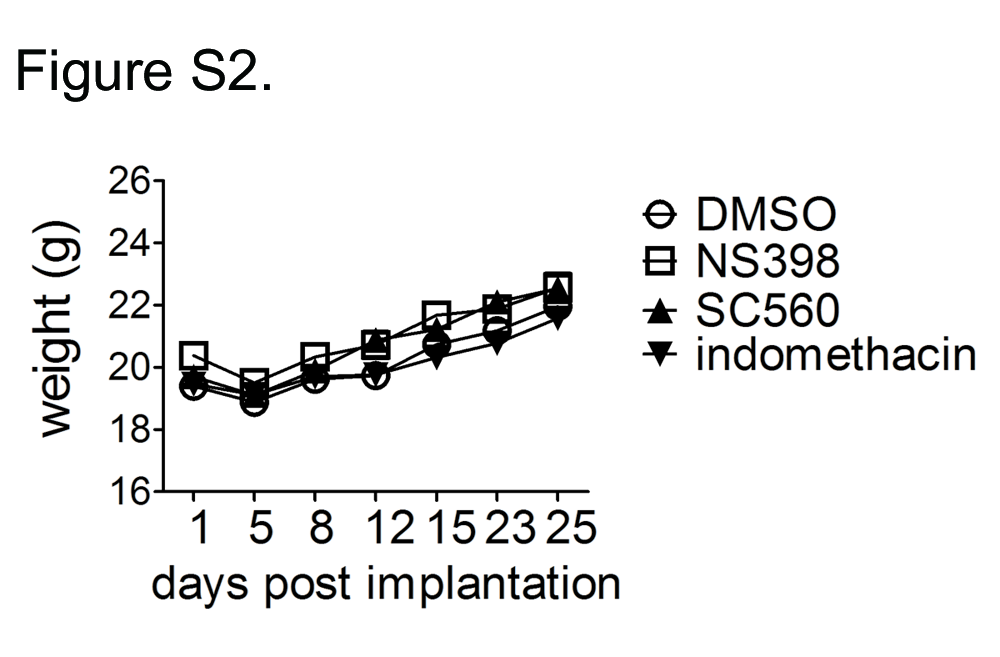

Supplement: S2 Fig — Female BALB/c mice were anesthetized and implanted subcutaneously with an osmotic pump carrying DMSO, SC-560, NS-398 or indomethacin. Mice weights were recorded twice per week for 1 month. Means ± SE, n = 5. (TIF) [file pone.0118203.s002.tif]

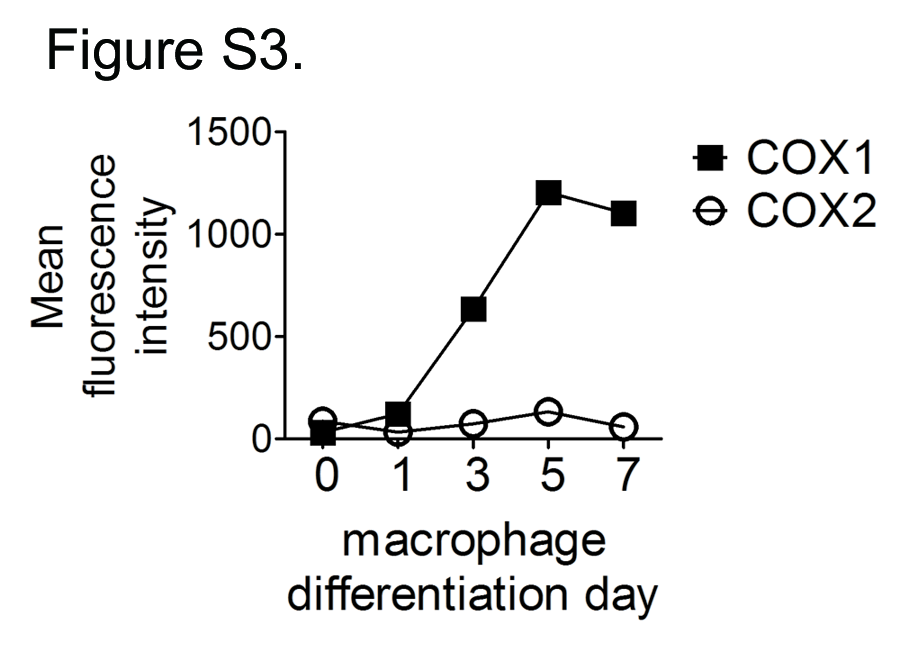

Supplement: S3 Fig — Bone marrow cells (Day 0) and differentiating macrophages (Day 1, 3, 5, 7) were fixed with 4% PFA, permeabilized and stained with COX-1 and COX-2 antibodies. Mean fluorescence intensities were analyzed using FACS. (TIF) [file pone.0118203.s003.tif]
